# Y-Chromosome Diversity in Modern Bulgarians: New Clues about Their Ancestry

**DOI:** 10.1371/journal.pone.0056779

**Published:** 2013-03-06

**Authors:** Sena Karachanak, Viola Grugni, Simona Fornarino, Desislava Nesheva, Nadia Al-Zahery, Vincenza Battaglia, Valeria Carossa, Yordan Yordanov, Antonio Torroni, Angel S. Galabov, Draga Toncheva, Ornella Semino

**Affiliations:** 1 Department of Medical Genetics, Medical University of Sofia, Sofia, Bulgaria; 2 Dipartimento di Biologia e Biotecnologie “L. Spallanzani”, Università di Pavia, Pavia, Italy; 3 Institute of Experimental Morphology and Anthropology with Museum, Bulgarian Academy of Sciences, Sofia, Bulgaria; 4 The Stephan Angeloff Institute of Microbiology, Bulgarian Academy of Sciences, Sofia, Bulgaria; IPATIMUP (Institute of Molecular Pathology and Immunology of the University of Porto), Portugal

## Abstract

To better define the structure and origin of the Bulgarian paternal gene pool, we have examined the Y-chromosome variation in 808 Bulgarian males. The analysis was performed by high-resolution genotyping of biallelic markers and by analyzing the STR variation within the most informative haplogroups. We found that the Y-chromosome gene pool in modern Bulgarians is primarily represented by Western Eurasian haplogroups with ∼ 40% belonging to haplogroups E-V13 and I-M423, and 20% to R-M17. Haplogroups common in the Middle East (J and G) and in South Western Asia (R-L23*) occur at frequencies of 19% and 5%, respectively. Haplogroups C, N and Q, distinctive for Altaic and Central Asian Turkic-speaking populations, occur at the negligible frequency of only 1.5%. Principal Component analyses group Bulgarians with European populations, apart from Central Asian Turkic-speaking groups and South Western Asia populations. Within the country, the genetic variation is structured in Western, Central and Eastern Bulgaria indicating that the Balkan Mountains have been permeable to human movements. The lineage analysis provided the following interesting results: (i) R-L23* is present in Eastern Bulgaria since the post glacial period; (ii) haplogroup E-V13 has a Mesolithic age in Bulgaria from where it expanded after the arrival of farming; (iii) haplogroup J-M241 probably reflects the Neolithic westward expansion of farmers from the earliest sites along the Black Sea. On the whole, in light of the most recent historical studies, which indicate a substantial proto-Bulgarian input to the contemporary Bulgarian people, our data suggest that a common paternal ancestry between the proto-Bulgarians and the Altaic and Central Asian Turkic-speaking populations either did not exist or was negligible.

## Introduction

Bulgaria is situated in the eastern part of the Balkan Peninsula, on the shores of the Black Sea linking Southeastern Europe to the Eurasian steppe as well as Anatolia and the Aegean islands. It lies on the postulated pathway that introduced modern humans into Europe in the Upper Paleolithic as attested by the series of assemblages at Bacho Kiro and Temnata Dupka Caves, which are considered signs of the Aurignacian culture expansion ∼ 40 kya [1]–[3]. It appears to have remained habitable even during the Last Glacial Maximum (LGM) [4] fuelling different expansion routes of post-glacial re-colonization. The presence in Bulgaria of some of the earliest farming sites, as well as some of the earliest evidence of copper metallurgy in Europe, indicates that this area also played a significant role in the Neolithic spread. On the other hand, the Kurgan expansion, triggered by the adoption of pastoral nomadism by peoples in the Pontic Steppes and by the domestication of the horse, spread into the Balkans in three waves ∼ 4 ky BC through Bulgarian territory [5].

In terms of history, the earliest among well-documented civilizations that inhabited present-day Bulgaria are the Thracians whose cultural legacy is still evident in the modern country, especially in its southern part. Two other populations playing an important role in the Bulgarian ethnogenesis were the Slavs and the proto-Bulgarians, who arrived almost simultaneously in the Early Middle Ages.

Until recently, it was considered that proto-Bulgarians, who founded the Danubian (Asparukh) Bulgaria (ancestor of the present-day Bulgaria) in the late 7th century AD, were a sparse Turkic population, which took the leadership and ruled over much more numerous Slavic populations living in the same area. From this scenario arose the notion that proto-Bulgarians did not play a significant role in the formation of the gene pool of contemporary Bulgarians.

The initial homeland of the proto-Bulgarians, called Balhara by Indians and Bactria by Greeks, was situated in the foothills of Pamir and Hindu Kush Mountains. In antiquity and the early Middle Ages, proto-Bulgarians founded three large and stable states in Europe: the first one was called “Old Great Bulgaria” (Η Παλαιά Μεγάλη Βουλγαρία) by the scholars of the Eastern Roman Empire and was situated in the area delimited by the Caucasus, the Caspian Sea and Dnieper River. Subsequently but almost simultaneously two other large states were established:Volga-Kama Bulgaria and Asparukh (Danubian) Bulgaria [6]–[12].

In their states, proto-Bulgarians built a number of towns-fortresses (Bulkhar-Balkh in the East Caucasus, Bilar and Bolgar on the Volga, Bolgarfehervar north of the Danube, and Pliska, the first capital of Danubian Bulgaria) as well as a defensive wall connecting the Danube and the Black Sea called “The Great Wall” [6], [8], [9], [11], [12]. Proto-Bulgarians were not only founders and organizers of state and unique military power, but they were also carriers of a developed civilization embracing the economy and artistic culture [8], [10], [11]. On the other hand, it is well known that at the time of the Danubian Bulgaria foundation Slavic tribes in the Balkans did not have any significant state and military organization [13].

Novel analyses of proto-Bulgarians epigraphic monuments, especially, of the major historical inscription – “the List of the Bulgarian Khans” - have revealed that the proto-Bulgarian language did not belong to the Turkic linguistic family. Therefore, leading turkologists [14]–[16] do not consider proto-Bulgarians a Turkic people, as also attested by the adoption of distinctive calendar systems by the two groups [17]. Differently, for grammatical features the proto-Bulgarian language gravitates towards the Pamir languages of the East Iranian group, which belong to the Indo-European branch. Despite its Slavic basis, the contemporary Bulgarian language contains many traces of the proto-Bulgarian language such as the kinship terminology system, an abundance of doublets for the same notion, a series of names of body parts, objects of material culture, and a great number of verbs and adjectives that are not found in either Slavic or Turkic languages, but are widely present in the Pamir languages [6], [10], [18].

One of the most important discoveries about Bulgarian history was recently published [10], [13], [19]. It shows that an entire array of previously unknown sources written in four languages - Old Bulgarian, Greek, Old German and Hebrew-Khazar - unanimously describe proto-Bulgarians as a quite numerous people [20]. In all of these sources proto-Bulgarians were referred to using similar expressions: “countless” [21], “too big and enormous population” (The Miracles of St. Demetrius of Thessaloniki), “numerous as the sand by the sea” (the letter from the Kagan of Khazaria Joseph; 961 AD), “so numerous that they did not need to build fortresses for defence” (“The Bavarian Geographer”) [20]. Some similar expressions were used even by the early Bulgarian rulers Omurtag and Presian [22]. Archaeological investigations have recently revealed that proto-Bulgarians represented a substantial part (evaluated in at least 32% [23] and even 60% [13], [19]) of the early Bulgarian population. An indirect indicator of the numerousness of proto-Bulgarians was the great number of their victorious war campaigns over the powerful Eastern Roman Empire (including the crushing defeat of Byzantium by Asparukh in 680 AD [20]) and the victory over the Arabs in 716 AD, which besieged Constantinople. Only a state with an army exceeding 100,000 soldiers was capable for such attacks. Obviously, the studies mentioned above suggest that proto-Bulgarians played a substantial role in the formation of the Bulgarian people.

From a genetic point of view, recent analyses of mtDNA [24] and autosomal [25], variation locate modern Bulgarians between Eastern European and Mediterranean populations. In particular, almost the entire Bulgarian mtDNA pool has West Eurasian origin and includes signature lineages of all the European peopling events from the Upper Paleolithic colonization of Europe to the more recent onset of the Neolithic in Europe [24]. Scarce information is however available for the patrilineal Bulgarian gene pool that, for its holoandric transmission, could retain signs of male mediated past migrations not necessarily detectable by mtDNA. To address this deficiency, we have conducted a high-resolution biallelic marker analysis of 808 Y chromosomes from contemporary Bulgarians, followed by a survey of microsatellite variation within the most informative haplogroups (Hgs). The observed diversity patterns were analyzed at both macro- and micro-geographic levels. This allowed us to establish a link between Y-chromosome lineages in modern Bulgarians and known prehistoric and historic events.

## Materials and Methods

### Sample Collection

We genotyped a total of 808 DNA samples from unrelated Bulgarian males. All of them gave informed consent and provided personal genealogical information prior to the sampling. Only individuals whose fathers were of Bulgarian origin and were born in the country were included in the study. The analyzed samples are a subset of those previously investigated for mtDNA variation [24].

**Figure 1:**
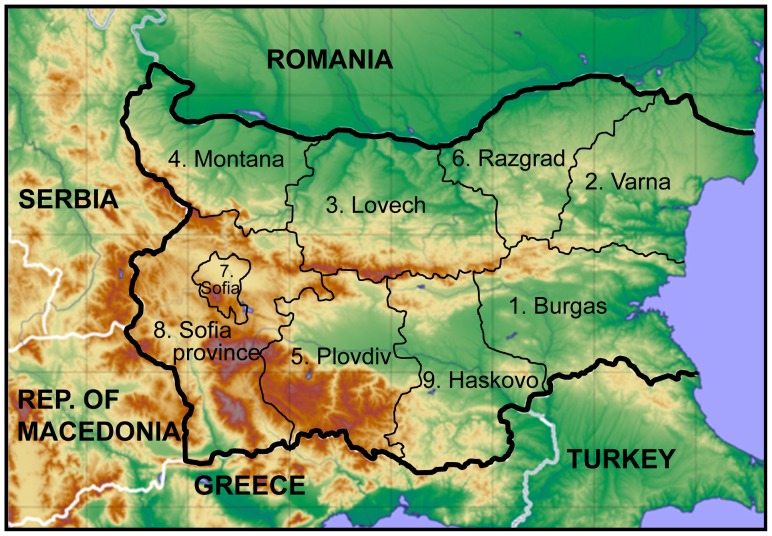
Map of Bulgaria outlining the former administrative provinces within which samples were allocated. The provinces are indicated as follows: 1. Burgas; 2. Varna; 3. Lovech; 4. Montana; 5. Plovdiv; 6. Razgrad; 7. Sofia City; 8. Sofia Province and 9. Haskovo.

In accordance with a comprehensive anthropological study of Bulgarians [26] we classified the samples according to the former (before 1999) administrative subdivision of the country (Figure 1). Thus, based on information on the paternal birthplace, 739 of the samples were unambiguously assigned to one of the nine provinces of Bulgaria, namely: Burgas (N = 45), Haskovo (N = 41), Lovech (N = 62), Montana (N = 80), Plovdiv (N = 159), Razgrad (N = 21), Sofia city (N = 59), Sofia province (N = 257), Varna (N = 15).

#### Ethics statement

This research has been approved by the Ethics Committee for Clinical Experimentation of the University of Pavia, Board minutes of the 5^th^ of October 2010. Geographical and genealogy information were ascertained by interview after having obtained their written informed consent.

### Genotyping

**Figure 2:**
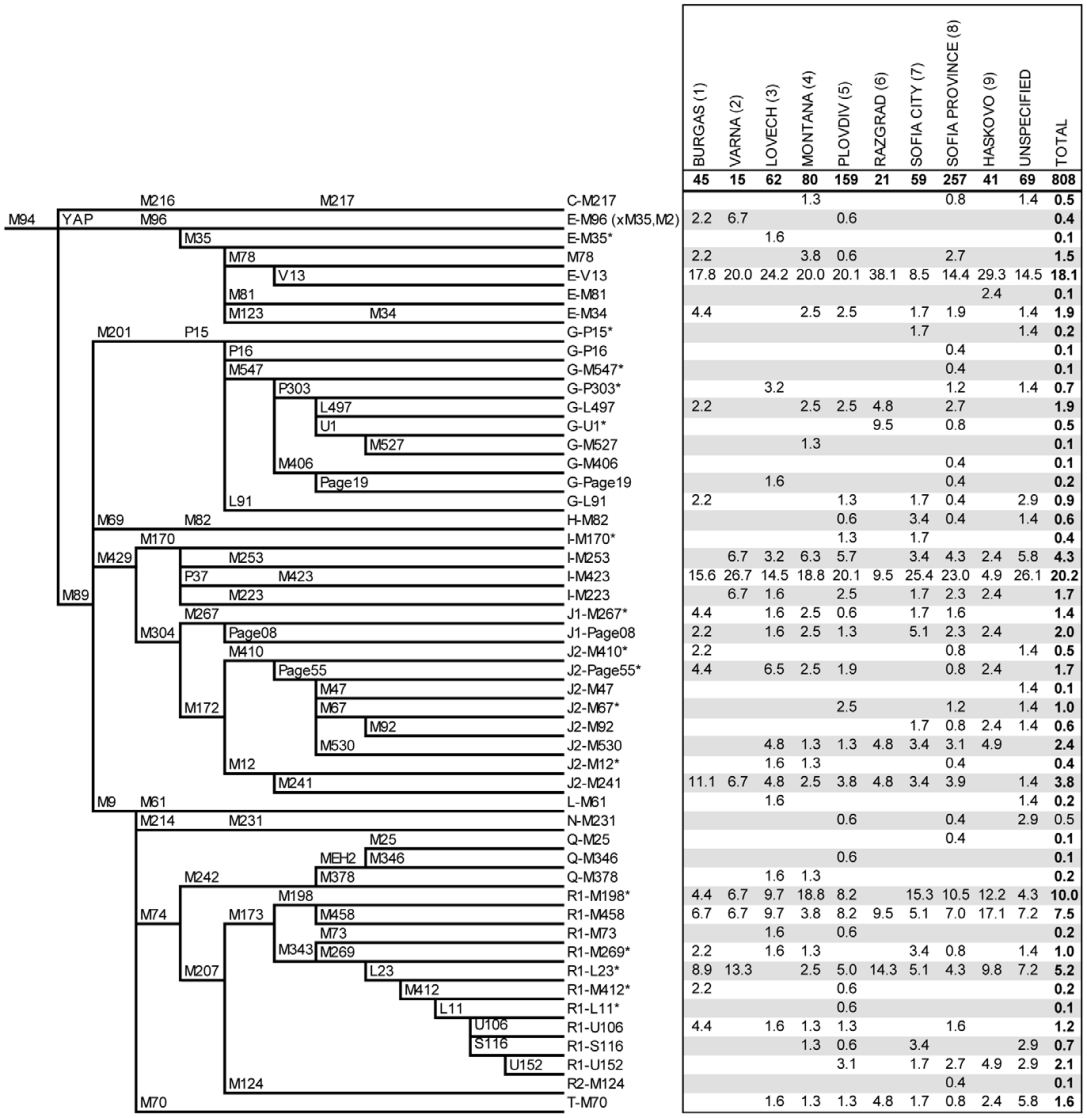
Phylogenetic relationships and percent frequencies of Y-chromosome haplogroups in each of the former provinces and in Bulgarians as a whole. The following markers: M2 (within Hg E), M365, M367, M368, M390 (within Hg J), M120, M323 (within Hg Q) and M529 (within Hg R) were typed but not observed.

In order to identify the haplogroups characterizing the Bulgarian male gene pool, we analyzed 75 biallelic markers. Markers M89 and M9 were analyzed in all samples; signature markers of haplogroups and sub-haplogroups (see phylogeny of Figure 2) where analyzed in a hierarchical way. Their genotyping was performed in hierarchical order, following the latest Y-chromosome phylogeny [27]. The YAP *Alu* insertion was analyzed by amplicon size detection, whereas all other markers were genotyped by PCR/RFLP, PCR/DHPLC assay or direct sequencing (Table S1). Haplogroups were labelled according to the mutation-based nomenclature rules proposed by the Y Chromosome Consortium [28].

Haplotyping was mainly performed on samples belonging to haplogroups E-V13, I-M423, J-M241, R-M458, R-L23*, R-U106 and R-U152. The following 17 Y-STR *loci*: DYS19, DYS385 a/b, DYS389I/II, DYS390, DYS391, DYS392, DYS393, DYS437, DYS438, DYS439, DYS448, DYS456, DYS458, DYS635 and Y-GATA H4 were amplified using the multiplex AmpFl/STR Y Filer Kit (Applied Biosystems) and read on ABI 310 genetic analyzer with GeneMapper software.

### Statistical Analyses

Principal Component Analyses (PCA) were performed to produce two-dimensional plots displaying the relationships between Bulgarians and other populations, as determined by Y-chromosome haplogroup frequencies. The PCA analysis was carried out using Excel through XLStat add-in.

The evaluation of the genetic structure in Bulgaria was achieved by analysis of molecular variance (AMOVA) [29] carried out with Arlequin 3.5 software [30]. In addition to the inter- and intra-regional variance analysis, two more AMOVA analyses were performed by subdividing the Bulgarian samples according to geographical and physico-geographical criteria. The geographic subdivision into Western, Central and Eastern Bulgaria, which turned out to be the most informative (with significant variance among groups), was adopted to evaluate the haplogroup STR variation used to estimate the upper bounds of expansion times.

Time estimates were performed only when more than five observations per population/region were available. Ages based on microsatellite variation within binary haplogroups were defined by the methodology of Zhivotovsky *et al.* [31] as modified according to Sengupta *et al.* [32]. In particular, it was estimated as the average squared difference in the number of repeats between all current chromosomes and the median haplotype, averaged over microsatellite loci and divided by the effective mutation rate of 6.9×10^−4^ per 25 years, with the SE computed over loci [31] (details, in Appendix A of Sengupta et al. [32]. A microsatellite evolutionary effective mutation rate of 6.9×10^−4^ per generation (25 years) was used [31] since it is suitable for use in situations where the elapsed time frame is ≥1000 years or ∼ 40 generations [33], appropriate given the prehistoric time depths explored in this study. It is worth mentioning that ambiguities related to past episodes of population history (e.g.: size fluctuations, bottlenecks, etc) create inherent uncertainties in the calibration of the Y-STR molecular clock, thus the estimated ages of microsatellite variation should be considered with caution.

In order to make relevant comparisons with previously analyzed populations/regions [34]–[37] haplogroup age estimates were (re-) calculated on a unified set of STR loci. In particular, analyses of STR variation within haplogroups were based on eight STR *loci* (DYS19, DYS389I, DYS389II, DYS390, DYS391, DYS392, DYS393 and DYS439), with the exception of Hg R-M458, for which the STR profiles were further reduced to seven *loci* (DYS19, DYS389I, DYS389II, DYS390, DYS391, DYS392 and DYS439).

Phylogenetic relationships of STR haplotypes within haplogroups I-M423, E-V13, R-M458, R-L23* and J-M241 were depicted by constructing median-joining (MJ) networks [38] using the Network 4.5.1.6 program (www.fluxus-engineering.com). The networks were calculated after having processed the data with the reduced-median method [39] and after having weighted the STR *loci* proportionally to the inverse of the repeat variance.

## Results and Discussion

### Haplogroup Frequency Distribution

From the total of 75 binary markers genotyped, 50 turned out to be informative. The most parsimonious relationships and the frequencies of the corresponding haplogroups are presented in Figure 2.

Western Eurasian haplogroups were found to encompass almost the entire Bulgarian Y-chromosome pool. Contributions from Central Asia (Hg C-M217) [40], [41], Northern Eurasia (Hg N-M231) and South West Asia (Hg Q-M242 derivatives, Hg L-M61 and Hg R-M124) [32], [42] were detected at almost negligible frequencies.

The most prevalent haplogroups in Bulgarians are I-M423 (20.2%) and E-V13 (18.1%). They represent the autochthonous and nearly endemic sub-clades of I-P37 and E-M78 in Southeastern Europe, respectively [34], [43]. Third in frequency is the common Eurasian haplogroup R-M17, which was found in 17.5% of Bulgarians, with 42.9% of them belonging to the European specific R-M458 sub-clade [36]. Haplogroup R-L23*, the eastern branch of the western Eurasian R-M269 haplogroup [37], relates the paternal ancestry of 5.2% of Bulgarians, representing nearly half of the M269 derived Y chromosomes. Next in frequency is Hg I-M253 (4.3%), which accounts for the majority of haplogroup I-M170 individuals in Northern Europe [44], [45]. It is followed by two J-M172 sub-branches, namely J-M241 and J-M530, observed at a frequency of 3.8% and 2.4%, respectively. The rest of the phylogenetically terminal haplogroups harboured frequency values of less than 2%.

### Y-chromosome Haplogroup Structure of Bulgarians at Macro- and Micro-Geographic Scales

In order to investigate the position of the Bulgarian population in the Euro-Asiatic context, PC analyses on haplogroup frequencies were carried out using informative literature data normalized to the highest possible binary phylogenetic resolution.

**Figure 3:**
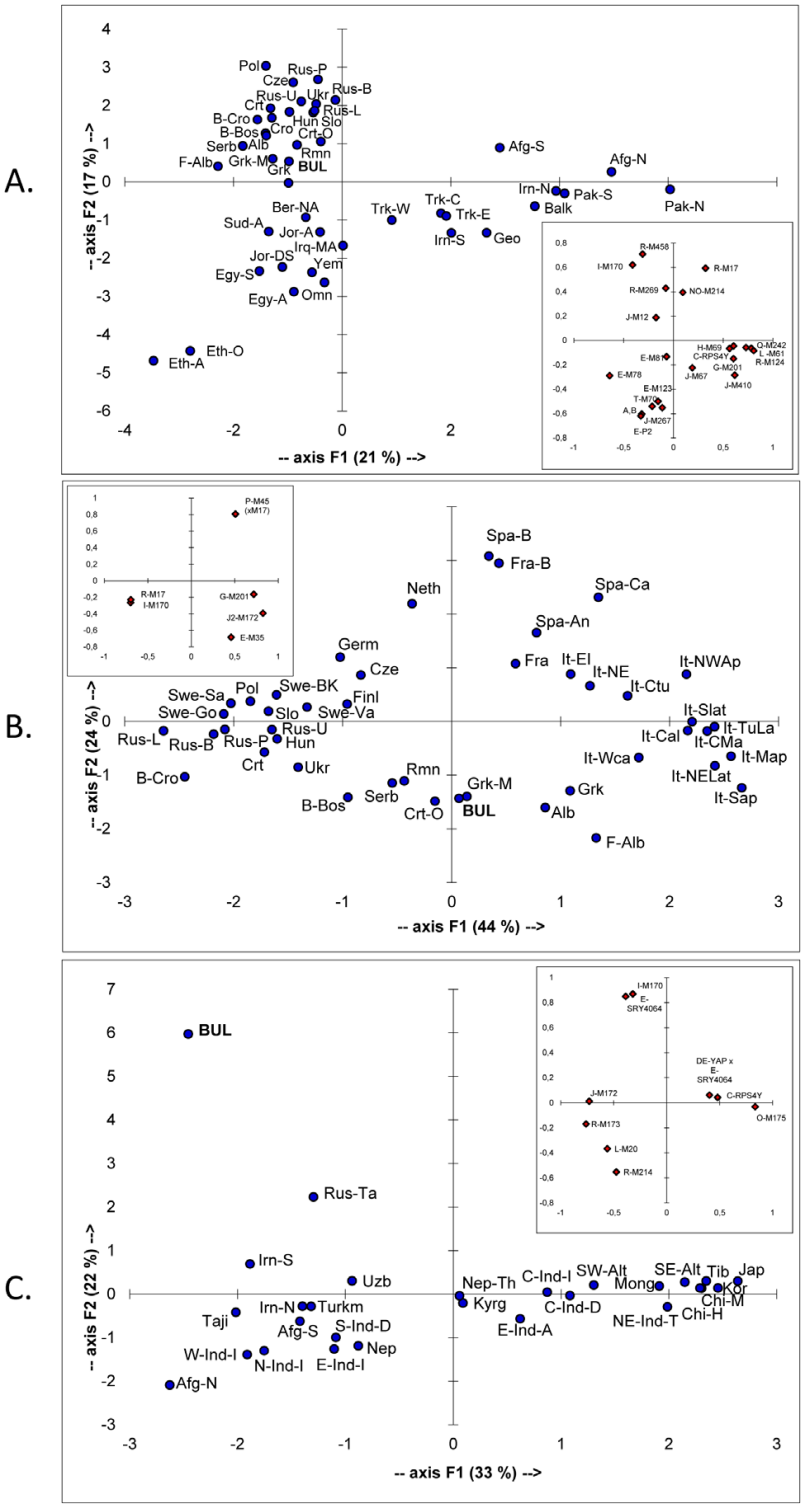
Principal components plots based on Y-chromosome haplogroup frequencies. (A) African and Eurasian populations analyzed at the highest level of phylogenetic resolution, (B) Bulgaria in the European context, at a lower level of phylogenetic resolution, and (C) Bulgaria in the Asian context, based only on informative Asian markers. Data and abbreviations are provided in Tables 2–4. Numbers in brackets indicate the proportion of the total genetic information retained by a given PC. Inset plot illustrates the contribution of each haplogroup.

The first PC analysis was conducted on haplogroup frequencies in Eurasian and African populations analyzed at the same high level of phylogenetic resolution reached in this survey (Table S2). The resulting PCA plot (Figure 3A) distinguishes four main clusters of populations: East Africans, Europeans, North African and Middle Eastern Arabic populations, and a South West Asian cluster formed by Turks, Iranians, Caucasus populations and Pakistanis. As shown in the inset plot, which illustrates the contribution of each haplogroup, the longitudinal separation along the first PC is mainly ascribable, on one side, to Hg I-M170 (almost restricted to Europe) and Hg E-M78 (frequently occurring in East Africa and Southeast Europe) and, at the opposite extreme, to Hgs L-M61 and R-M124 (frequent in South Asia). The second PC separates populations by latitude, as Hgs R-M17 and R-M458 are most frequent in Europeans, Hgs A-M91, B-M60 and E-P2 are almost exclusive to East African groups and Hgs E-M123 and J-M267 are most frequent in North Africa and the Southern Middle East. Europeans separate from Turks, Caucasus populations, Iranians and Pakistanis along the PC1 and from Arabic and East African populations along the PC2. In the plot of this analysis, Bulgarians distribute within the European cluster, very close to Macedonian Greeks, but relatively far from their south-eastern neighbours - the Turks. The affinities shown in Figure 3A corroborate those displayed in a genome-wide PC analysis involving several hundred subjects (including 13 Bulgarians) genotyped at several thousand genome autosomal SNPs [25].

In the other two PC analyses the position of the Bulgarian Y-chromosome gene pool was investigated in the European (Figure 3B) and Asian (Figure 3C) contexts, through the analysis of the most frequent European (Table S3) and Asian (Table S4) haplogroups, respectively.

In the plot of Figure 3B, the distribution of the populations along the PC1 reflects the prevalence of Hg I-M170 in Scandinavia and the Eastern Adriatic, the predominance of Hg R-M17 in North-Central and Northeastern Europe and the increasing frequency of haplogroup J-M172 towards Italy and the Southern Balkans. The separation along the second component, is mainly due to Hgs E-M35 and P-M45(xR-M17). The first is most represented in the Southern Balkans; the second is most frequent among Western European populations and, based on previous studies [37], [46], [47] is most probably overwhelmingly represented by haplogroup R-M269. This analysis confirms the position of Bulgarians close to Macedonian Greeks and in the proximity of Slavic populations from the Western Balkans, which is not the case with the remainder of the Slavic populations considered.

In the plot of Figure 3C, the populations are distributed in a longitudinal pattern generated essentially by the PC1. This separation is determined by the prevalence of the East Asian Hg O-M175 and the Central Asian Hg C-RPS4Y in the east and the increasing frequencies of Hgs J-M172 and R-M173 in the west. The PC2, which is mainly due to Hgs I-M170 and E-SRY4064, clearly separates Bulgarians from the compared populations. On the whole, Bulgarians are distant from Altaic populations and populations residing in the north of the Pamir region and they are also distant from Kazan Tatars and Iranians, although to a lesser extent.

The geographical partitioning of genetic diversity in Bulgarians was investigated by AMOVA, the results of which are listed in Table 1. The variation among populations from the former administrative provinces was small and of uncertain significance (0.38%, p≈0.05); no significant results were obtained when the samples were grouped into physico-geographical regions – the Black Sea coast, the Danubian Plain, Thrace and the southwest mountainous region, and only barely significant variation among groups (0.49%, p≈0.05) was unexpectedly revealed when the samples were assigned to three broader geographical regions - Eastern, Central and Western Bulgaria. These findings show that the modern Bulgarian Y-chromosome gene pool is rather homogenous and the Balkan Mountains probably did not act as a strong barrier to gene flow.

**Table 1:** Analysis of molecular variance in populations of the former nine provinces of Bulgaria.

	Source of variation	Variance components	Percentage of variation
*No grouping*			
	Among populations	0.00173*	0.38
	Within populations	0.44967	99.62
*Grouping into Eastern, Central and Western Bulgaria (provinces 4, 7* *and 8 vs provinces 3, 5 and 9 vs provinces 2, 6 and 1)*			
	Among groups	0.00221*	0.49
	Among populations within groups	0.00014	0.03
	Within populations	0.44967*	99.48
*Grouping into physico-geographical regions (provinces 1 and 2 vs* *provinces 7 and 8 vs provinces 6, 3 and 4 vs provinces 5 and 9)*			
	Among groups	0.00088	0.19
	Among populations within groups	0.00097	0.21
	Within populations	0.44967	99.59

* p≈0.05.

### Y-STR Variation and Diffusion Patterns of the Main Haplogroups

Subsets of Bulgarians samples belonging to the most frequent haplogroups were further analyzed for 17 STR loci (Table S5) and the obtained results were used to investigate time upper bounds and diffusion patterns.

The networks of the haplotypes (Table S6) associated with haplogroups I-M423, E-V13, R-M458, R-L23*, J-M241 in Bulgaria and in informative populations/regions are illustrated in Figure S1, whereas their relative coalescence times are reported in Table S7. Important caveats to consider are the inherent uncertainties in the calibration of the Y-STR molecular clock and the inflation of the time estimates caused by rare outlier alleles and multiple founders or recurrent gene flows during population formation.

The network of haplogroup I-M423 is characterized by a star-like shape centred on the most frequent haplotype, present in all Balkan populations. The network topology, together with the age estimates, is in accordance with previous inferences that haplogroup I-M423 is the genetic record of Balkan Mesolithic foragers and their expansion after the adoption of agriculture [34]. It is worth noticing that the Bulgarian samples are scattered all over the network; belonging to expanded, rare, and unique haplotypes. This diversity is consistent with an associated antiquity of Hg I-M423 in Bulgaria.

Haplogroup E-V13 displays a star-like network radiating from a central haplotype mainly found in the Balkan populations. This pattern, together with coalescence estimates, points to a recent and rapid expansion of this lineage in the Balkans. Not considering Bosnian Croats and Macedonian Greeks, for which standard errors are too large, the highest age in the Balkans, dating back to Mesolithic times, is found in Western Bulgaria (9.3±3 kya). This value, which overlaps that registered in Turkey (10.6±3 kya), indicates that haplogroup E-V13 was already present (if not originated) in Mesolithic times in Western Bulgaria from where it underwent expansion with the transition to farming.

The network of Y-STR haplotypes associated with Hg R-M458, the European branch of haplogroup R-M17/M198, is characterized by a star-like center of expansion and complex reticulations that can be solved only by improving the phylogenetic resolution of this haplogroup. Still, the majority of the M458 derived samples occupy the star-like portion of the network, in agreement with a North-Central European origin and the subsequent expansion previously reported [36]. Although it is likely that the age estimates of this lineage mainly reflect these demographic events, it is not possible to exclude that they are biased by the coexistence of different sub-lineages within this unresolved haplogroup. In this regard, it is worth mentioning that, as previously suggested [48]–[50], haplogroup R1a-M17 could be a signal of various events ranging from early post-LGM expansions to more recent Slavic demography. Hence, the old coalescent times, such as those obtained for Eastern Bulgaria (12.4±5 kya) and the Caucasus (10.1±3 kya), should be considered with caution in that multiple founders (or multiple demographic episodes) can inflate these estimates.

The network of Hg R-L23* is characterized by multiple reticulations, which confirm that this haplogroup includes sub-clades yet to be discovered [37]. The frequency and variance distributions of R-L23 (data not shown), together with its age variation, locate the most ancient presence of this lineage in the Circum-Pontic region, where similar estimates, coinciding with the post-glacial period, are registered: 16.8±7 kya in Eastern Bulgaria, 14.3±1 kya in Romania, 14.0±3 kya in the Caucasus and 13.6±2 kya in Anatolia. We abstain from premature conclusions on the coalescent estimate in Eastern Bulgaria since a significant portion of this value derives from a very different singleton haplotype whose exclusion substantially decreases the age estimate to 9.3±4 kya.

Haplogroup J-M241 shows a network with the central and most frequent haplotype being widespread in the Southern Balkans - a likely consequence of a rapid expansion probably started in Neolithic times in Asia Minor [34]. Since the periphery of the network is mainly occupied by haplotypes found outside this region (Apulians, Indians and Nepalese) the present results do not provide any useful evidence for the identification of the J-M241 homeland. On the other hand, the high age estimates in these populations could be due to recurrent gene flow from different sources. Leaving aside Apulians, Indians and Nepalese, the highest ages, compatible with a Neolithic expansion, are obtained in regions around the Black Sea, namely Anatolia (9.1±2 kya) and Bulgaria, in particular its central part (7.8±3 kya). Consequently, in this region, haplogroup J-M241 can be considered as a genetic signal of the expansion of farmers towards Southeast Europe possibly enhanced by the breaching of the Bosphorus Sill and the flood of the Pontic Lake with marine water.

### Conclusions

In the present study we assessed the male-mediated genetic legacy in modern Bulgarians by analyzing their Y-chromosome composition and by surveying the internal variation within the main haplogroups. We found that a major part of the Bulgarian Y-chromosome gene pool is constituted by Western Eurasian haplogroups with a particular affinity to neighbouring groups from the Balkans and Greece, in agreement with previous anthropological [26] and mtDNA studies [24]. When analyzed in a broader context, the Bulgarian haplogroup profile is located among European populations and apart from Altaic and Central Asian Turkic-speaking populations.

Within the country, the male genetic variation is structured among Western, Central and Eastern Bulgaria, rather than among the physico-geographical regions (the Black Sea coast, the Danubian Plain, Thrace and the Southwest mountainous region). This pattern of genetic partitioning indicates that the Balkan Mountains have been permeable to human movements.

Interesting results from the lineage analysis can be summarized as follows: (i) R-L23*, the eastern branch of haplogroup R-M269, is present in Eastern Bulgaria since the post glacial period; (ii) haplogroup E-V13, which probably originated in Western Asia, has a Mesolithic age in Bulgaria from where it expanded after the spread of farming marked by haplogroup G-P15, J-M410 representatives; (iii) haplogroup J-M241 probably reflects the Neolithic westward expansion of farmers from the earliest sites along the Black Sea.

In addition, an important consideration arises from the finding that haplogroups C-M217, N-M231 and Q-M242, which are common in Altaic and Central Asian Turkic-speaking populations [40], [41], occur at the negligible frequency of only 1.5% in modern Bulgarians. This observation is in agreement with the results of recent linguistic studies which demonstrated that the proto-Bulgarian language does not belong to the Turkic family but it relates to the Indo-European languages of the East Iranian group, whose traces still persist in the modern Bulgarian language, despite its Slavic basis. Thus, taking into account the novel and detailed historical studies indicating that proto-Bulgarians were quite numerous (32% or perhaps even 60% of the population in early Danubian Bulgaria) [6]–[13], [19], [23], it follows that a shared paternal ancestry between proto-Bulgarians and Altaic and Central Asian Turkic-speaking groups either did not exist or was negligible.

## Supporting Information

Figure S1 **Median-joining** ***networks*** for haplogroups I-M423, E-V13, R-M458, R-L23* and J-M241. For each network, circles and colored sectors are sized according to the number of subjects sharing the haplotype, as the smallest circles and sectors represent one subject. The lengths of the connecting lines are proportional to the number of mutational steps separating two haplotypes.
(TIF)

Table S1 **Details concerning the examined biallelic markers.**
(XLSX)

Table S2 **Absolute frequencies of Y-chromosome haplogroups and subhaplogroups in the 40 populations included in the PCA of** **figure 3A** **.**
(XLSX)

Table S3 **Absolute frequencies of the main Y-chromosome haplogroups in the 44 populations included in the PCA of** **figure 3B** **.**
(XLSX)

Table S4 **Absolute frequencies of Y-chromosome haplogroups in the 27 populations included in the PCA of** **figure 3C** **.**
(XLSX)

Table S5 **Y-STR haplotypes (17 loci) observed in the Bulgarian samples analyzed.**
(XLSX)

Table S6 Y-STR data used to assess the variation within haplogroups E-V13, I-M423, J-M241, R-L23*, R-M458, R-U106 and R-U152.
(XLSX)

Table S7 Age estimates of microsatellite variation and Standard Error within haplogroups E-V13, I-M423, J-M241, M458, R-L23*, R-U106 and R-U152 in Bulgarians and other Eurasian populations. The number of samples considered is given in parentheses.
(XLSX)
