# Supplementary figures and images for: Y-Chromosome Diversity in Modern Bulgarians: New Clues about Their Ancestry
Source: PLoS One. 2013 Mar 6;8(3):e56779. doi: 10.1371/journal.pone.0056779 (PMC3590186; doi:10.1371/journal.pone.0056779)

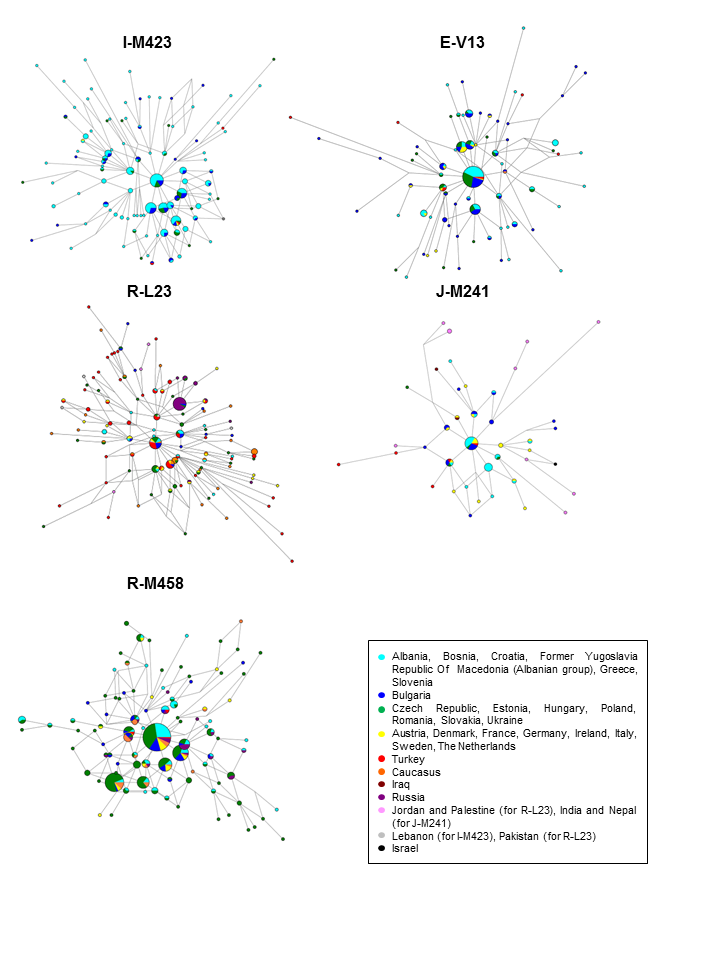

Supplement: Figure S1 — Median-joining networks for haplogroups I-M423, E-V13, R-M458, R-L23* and J-M241. For each network, circles and colored sectors are sized according to the number of subjects sharing the haplotype, as the smallest circles and sectors represent one subject. The lengths of the connecting lines are proportional to the number of mutational steps separating two haplotypes. (TIF) [file pone.0056779.s001.tif]
